# Pan-genome analysis of the *AP2/ERF* gene family and its functional characterization in banana plant height

**DOI:** 10.3389/fpls.2026.1944831

**Published:** 2026-10-19

**Authors:** Zhiwei Lu, Mingwei Li, Shiyu Lu, Weijun Xiao, Zhiwei Jia, Yulin Hu, Yajie Duan, Zhencai Pang, Dequan Sun, Xiaowan Hou, Huigang Hu

**Affiliations:** 1 State Key Laboratory of Tropical Crop Breeding, South Subtropical Crops Research Institute, Chinese Academy of Tropical Agricultural Sciences, Zhanjiang, China; 2 Key Laboratory of Postharvest Physiology and Technology of Tropical Horticultural Products of Hainan Province, Zhanjiang, Guangdong, China; 3 Key Laboratory of Hainan Province for Postharvest Physiology and Technology of Tropical Horticultural Products, Zhanjiang, Guangdong, China; 4 Chinese Academy of Agricultural Sciences, Beijing, China

**Keywords:** AP2/ERF family, banana, haplotype, pan-genome, plant height, transcription factor

## Abstract

**Introduction:**

Banana is a globally essential staple and industrial crop whose sustainable production is frequently challenged by excessive plant height and susceptibility to wind-induced lodging. Managing plant architecture is therefore critical for improving lodging resistance, yet the underlying genetic mechanisms governing plant height in banana remain poorly understood.

**Methods:**

To elucidate the genetic basis of plant height, we performed a systematic pan-genome analysis of the *AP2/ERF* gene family across 18 diverse banana genomes. The identified genes were characterized for their duplication mechanisms and selective pressures. Furthermore, we integrated the pan-genomic dataset with published phenotypic data using a haplotype-based association study to uncover key candidate genes and analyze the geographic distribution of their associated alleles across accessions.

**Results:**

A total of 4,732 *MaAP2ERF* genes were identified, yielding 468 non-redundant representative members. Dispersed duplication was the primary driver of family expansion (43.38%), whereas whole-genome duplication (WGD) established the conserved core foundation, accounting for all 42 core genes shared across all genomes. In contrast, dispersed duplication drove all 157 private genes, while the remaining 269 dispensable members arose from a combination of five duplication mechanisms. Evolutionary analysis indicated strong purifying selection on most members (K_a_/K_s_ ratios between 0.2 and 0.5). Haplotype-based association identified *MaAP2ERF144* and *MaAP2ERF407* as key candidates for plant height, with *MaAP2ERF407* demonstrating a dual association with stem elongation and lignin content for mechanical strength. Geographic analysis revealed that height-increasing alleles are mainly concentrated in Southeast Asia, Oceania, and East Africa, whereas dwarf alleles predominate in East Asian accessions.

**Discussion:**

These findings provide a comprehensive genomic framework for the banana *AP2/ERF* gene family and elucidate the evolutionary mechanisms shaping its diversity. The identified functional candidate genes and key haplotype loci offer valuable genetic targets for precision molecular breeding to develop high-yielding, lodging-resistant banana cultivars with optimized plant architecture.

## 1 Introduction

Banana (*Musa* spp.) represents the fourth most important staple food crop globally after rice, wheat, and maize, serving as a primary source of carbohydrates and income for over four hundred million people in tropical and subtropical regions (Arshad et al., 2025). Despite its immense economic and nutritional value, the sustainable production of banana is severely threatened by the excessive plant height of widely cultivated commercial varieties. Tall banana plants are particularly vulnerable to lodging caused by tropical storms and heavy winds, which frequently result in substantial economic devastation for growers (Wang et al., 2021). Furthermore, the height of these plants increases the labor intensity of harvesting and complicates the application of agricultural management practices, limiting the overall planting density and yield potential (Cai et al., 2023). Consequently, the identification of genetic factors that regulate plant architecture and the development of semi-dwarf varieties have become urgent priorities for modern banana breeding and genetic engineering efforts.

The determination of plant height is a sophisticated biological process governed by complex transcriptional networks and phytohormone signaling pathways (Wang et al., 2024; Zhao et al., 2024). Among the numerous transcription factor families involved, the APETALA2/Ethylene Responsive Factor (AP2/ERF) family has been identified as a critical regulator of plant architecture and organ development (Licausi et al., 2013; Feng et al., 2020). Early groundbreaking research in *Arabidopsis thaliana* established the SHINE clade of AP2/ERF transcription factors as essential regulators of cuticular wax accumulation and epidermal organization which collectively coordinate the development of plant protective layers (Aharoni et al., 2004). Subsequently, researchers utilized quantitative trait loci (QTL) mapping and genome-wide association studies (GWAS) in plants to uncover the pivotal role of this family in plant height development. For example, a multi-locus genome-wide association study in sorghum landraces identified a prominent AP2/ERF member as a strong candidate gene significantly associated with the genetic control of plant height (Wondimu et al., 2023). Additionally, map-based cloning in wheat demonstrated that the AP2/ERF transcription factor TaERF-A1 functions as a positive regulator of stem elongation where its natural variation confers a semi-dwarf and lodging-resistant architecture (Li et al., 2025a). Furthermore, the integration of meta-QTL analysis and association mapping in sesame most recently revealed that candidate genes involved in ethylene signaling, specifically from the AP2/ERF family, likely regulate plant architecture and height (Gebeyehu and Ortiz, 2026). Recent gene family analyses in various plant species have further confirmed that specific *AP2/ERF* lineages are evolutionarily specialized to either promote or suppress vertical growth, making them ideal targets for crop architectural modification (Feng et al., 2020).

The AP2/ERF transcription factor family is characterized by a highly conserved DNA-binding domain of approximately sixty amino acids. Based on the number of domains and additional sequence motifs, the family is traditionally subdivided into four major subfamilies which include the AP2, DREB, ERF, and Soloist groups (Nakano et al., 2006). Currently, the genomic identification and characterization of the banana *AP2/ERF* family is documented in only a single genome-wide investigation (Lakhwani et al., 2016). However, single-genome studies are inherently limited as they fail to account for the extensive genetic diversity and presence-absence variations (PAV) that exist among different *Musa* accessions and wild relatives. Complex traits like plant height are often influenced by lineage-specific gene expansion or loss events that cannot be fully represented by a single reference genome (Li et al., 2025a). Pan-genome and pan-gene family analyses have emerged as powerful tools to overcome these limitations by establishing a comprehensive and representative genomic architecture that integrates the genomic information of multiple cultivars (Bai et al., 2025; Sun et al., 2025). Applying a pan-gene family approach to the AP2/ERF family is therefore essential for resolving the comprehensive regulatory architecture and for accurately identifying the core and variable members that drive plant height variation across diverse banana populations.

In this study, we conducted a systematic pan-gene family analysis of the AP2/ERF family across eighteen diverse banana genomes, to establish a comprehensive framework for identifying key candidate genes associated with plant height variation. We first characterized these members by evaluating their protein properties, evolutionary relationships, and selection pressures. To understand the evolutionary drivers of the family, we analyzed the gene duplication modes and calculated the K_a_/K_s_ ratios to reveal the selection pressures acting on different subfamilies. Subsequently, we integrated our pan gene family results with published population phenotypic datasets through a haplotype based association study to identify the critical regulators of plant architecture. This approach identified *MaAP2ERF144* and *MaAP2ERF407* as lead candidate genes, with *MaAP2ERF407* demonstrating a dual association with both plant height and lignin content for mechanical strength. Further characterization of elite haplotypes across global accessions unveiled distinct geographical partitioning, reflecting the architectural diversity preserved across regional germplasms. Collectively, these findings establish a comprehensive genomic landscape of the banana *AP2/ERF* family and highlight promising candidate loci to engineer enhanced mechanical strength and desirable plant stature in cultivated germplasm.

## 2 Materials and methods

### 2.1 Pan-genomic identification and physicochemical characterization

Genome assemblies and protein sequences for eighteen representative Musaceae accessions were retrieved from the Banana Genome Hub (Droc et al., 2022, Droc et al., 2013). To ensure maximum genetic diversity, this set encompasses three genera: *Musa*, *Ensete*, and the monotypic genus *Musella*. Specifically, the collection includes *M. acuminata* (cv. ‘Baxijiao’, ‘Pahang’, and subspecies banksii, burmannica, zebrina, Pisang Madu), *M. balbisiana*, *M. beccarii*, *M. itinerans*, *M. schizocarpa*, *M. textilis* (two assemblies including ‘Abaca’), *M. troglodytarum* (two assemblies including ‘Utafun’), *Ensete glaucum*, *E. ventricosum*, and *Musella lasiocarpa*. Detailed information for each genome assembly is provided in **Supplementary Table S1**. To identify the *AP2/ERF* gene family, the Hidden Markov Model profile for the AP2 domain (PF00847) was obtained from the Pfam database (Mistry et al., 2021) and queried against the protein datasets of the 18 genomes using HMMER v3.3.2 with an *E*-value cutoff of ≤1×10^−5^ (Eddy, 2011). Supplementary BLASTP searches were further conducted against the 18 protein databases using verified AP2/ERF protein sequences as queries with an *E*-value threshold of ≤1×10^−5^ to ensure exhaustive retrieval of family members. All candidate sequences were subsequently validated for domain integrity through the NCBI Conserved Domain Database (Lu et al., 2020). To ensure that only functional, transcriptionally viable transcription factors were retained and to exclude pseudogenic relics or transposable element fragments prevalent in the *Musa* genome, only evidence-curated gene models with intact AP2 domains were retained, confirming a total of 4,732 valid AP2/ERF sequences across the 18 genomes. Following the validation of these 4,732 candidate MaAP2ERF sequences, a non-redundant set of representative members was extracted to accurately characterize the pangenome family. To achieve this, an orthogroup-based collapsing strategy was implemented using OrthoFinder (v2.5.4) (Emms and Kelly, 2019) to group sequences based on an all-versus-all BLASTP approach. Clustering was performed with a sequence similarity threshold of E-value < 1e ^−10^, a minimum protein identity of 50%, and a reciprocal sequence coverage of at least 70%. To designate a single representative for each orthogroup, a hierarchical selection priority was applied: members from the Musa acuminata ‘Baxijiao’ (BXH1) reference genome were prioritized; for orthogroups absent in BXH1, the member with the highest sequence integrity and longest protein length from the supplementary genomes (such as wild accessions) was selected. Through this strategy, 468 non-redundant representative members were ultimately identified from the total pool of 4,732 candidate genes. To characterize the genomic distribution of the family, these 468 members were categorized into two major groups: (1) core genes (N = 42), defined as those strictly conserved in all 18 analyzed genomes; and (2) variable genes (N = 426), which encompass dispensable genes (N = 269, present in 2 to 17 genomes) and private genes (N = 157, unique to a single genome assembly). Theoretical isoelectric points and molecular weights were predicted using the ProtParam tool on the ExPASy server (Duvaud et al., 2021). Proteins with an instability index below 40 are predicted as stable, while those exceeding 40 are classified as unstable (Guruprasad et al., 1990). GRAVY values reflect the overall hydropathy: negative values indicate hydrophilic (soluble) proteins, whereas positive values denote hydrophobic proteins (Kyte and Doolittle, 1982). Subcellular localization was predicted using the WoLF PSORT online server (Horton et al., 2007).

### 2.2 Phylogenetic analysis and subfamily classification

Multiple sequence alignments of 468 representative *MaAP2ERF* proteins and 121 known *Arabidopsis thaliana* AP2/ERF members were performed using ClustalW with default settings (Thompson et al., 1994). A phylogenetic tree was constructed using the Maximum Likelihood method in MEGA 11 with the JTT+G substitution model and 1,000 bootstrap replicates (Tamura et al., 2021). Subfamily classification was determined according to the phylogenetic topology and established nomenclature in *Arabidopsis* (Nakano et al., 2006).

### 2.3 Gene structure and duplication mode analysis

The exon-intron structures and intron numbers for each *MaAP2ERF* gene were determined by comparing coding sequences with their corresponding genomic sequences and were visualized using TBtools (Chen et al., 2020). To investigate the evolutionary drivers of family expansion, gene duplication modes were analyzed using the MCScanX toolkit integrated within TBtools (Wang et al., 2012). An all-against-all BLASTP search was performed with an *E*-value cutoff of ≤1×10^−10^ and the top 5 non-self hits retained (-evalue 1e^-10^ -max_target_seqs 5). Collinear blocks were identified using default MCScanX criteria (match_score = 50, gap_penalty = -1, match_size = 5, max_gaps = 25). Duplication modes were deterministically classified into five non-overlapping tiers based on the strict hierarchical priority embedded in MCScanX: whole-genome duplication (WGD)/segmental (≥5 collinear anchors), tandem (≤2 gene distance on the same chromosome), proximal (≤20 gene distance without collinearity), dispersed, and singleton. This hierarchical scheme deterministically eliminates ambiguous assignments across the representative members.

### 2.4 Selection pressure calculation

Nonsynonymous (Ka) and synonymous (Ks) substitution rates were calculated for duplicated *MaAP2ERF* gene pairs using the Simple Ka/Ks Calculator in TBtools based on codon-based alignments (Chen et al., 2020). To ensure rigorous estimates, only gene pairs with a reciprocal sequence coverage ≥ 70% and identity ≥ 50% were included. Gene pairs with Ks=0 (undefined division-by-zero), mutational saturation (Ks>2.0), or singletons lacking detectable duplication partners were excluded and denoted as ‘NA’. A total of 353 genes with valid Ka/Ks values were utilized for subsequent distribution modeling and statistical testing without artificial imputation. To evaluate the distribution of selective pressures across different duplication types and subfamilies, ridgeline density plots and boxplots were generated using the ggplot2 package in R software version 4.3.0 (Wickham, 2016). Statistical differences in Ka/Ks distributions among the five duplication types were evaluated using the non-parametric Kruskal-Wallis test followed by pairwise Wilcoxon rank-sum tests (Dunn’s test) with Benjamini-Hochberg FDR correction (*P* < 0.05). Genes with Ka/Ks ratios significantly greater than 1.0 were identified as candidates undergoing positive selection, while those with ratios between 0 and 1.0 were considered to be under varying degrees of purifying selection.

### 2.5 Phenotypic data collection and population characteristics

Plant height and lignin content data for the 155 and 184 banana accessions were obtained from previously published phenotypic datasets (Li et al., 2025b; Li et al., 2026). These accessions represent a diverse global collection. The phenotypic values used in our association study were recorded at the Danzhou Experimental Station in Hainan, China. We used measurements from this single site to ensure that all accessions were grown under consistent environmental conditions during the data collection process.

### 2.6 Haplotype-based association study

Raw population resequencing reads for the banana accessions used in this study were retrieved from the NCBI Sequence Read Archive (SRA) database under project PRJNA1178355, which were originally reported (Li et al., 2025b; Li et al., 2026). Quality control and adapter trimming were performed using fastp v0.20.1 with default parameters. The cleaned reads were aligned to the Musa acuminata ‘Baxijiao’ reference genome (BXH1) using BWA-MEM v0.7.17 with default settings (Li, 2013). This pan-reference was constructed by using the *Musa acuminata* ‘Baxijiao’ (BXH1) genome as the backbone and appending the unique genomic sequences of the 83 MaAP2ERF members identified from the other 17 banana genomes that were absent in BXH1. This strategy ensured a consistent coordinate system for variant calling across the entire family. Variant calling was performed following the GATK Best Practices workflow (v4.2.0). Briefly, genomic VCF (gVCF) files for each sample were first generated using HaplotypeCaller in ERC mode. These gVCFs were then consolidated into a GenomicsDB database via GenomicsDBImport and subsequently joint-genotyped using GenotypeGVCFs to produce a comprehensive population VCF file. To ensure high-quality variant calls, hard filtering was applied to the raw VCF file based on GATK Best Practices criteria: QualByDepth (QD) < 2.0, MappingQuality (MQ) < 40.0, FisherStrand (FS) > 60.0, and StrandOddsRatio (SOR) > 3.0. Subsequently, population-level variants were further refined using VCFtools (v0.1.16) to retain only biallelic SNPs with a minor allele frequency (MAF) > 0.05 and a minimum genotype call rate of 80% (missingness < 20%).

To perform the haplotype-based association analysis, genomic variations (SNPs and InDels) within the coding and promoter regions (2 kb upstream of the start codon) of each *MaAP2ERF* member were extracted from the population resequencing VCF files. Haplotypes were defined based on all non-synonymous SNPs and significant variations in the promoter regions using a custom Python script (haplotype_analysis_pipeline.py, provided as **Supplementary Material S1**). The association between distinct haplotypes and plant height/lignin content was statistically evaluated using one-way Analysis of Variance (ANOVA) followed by Tukey’s Honestly Significant Difference (HSD) test (P<0.05). To further ensure statistical robustness and prevent spurious associations driven by rare genotypes, haplotypes with a sample size of fewer than three accessions were excluded from the analysis. Accessions belonging to these minor haplotypes were excluded from the primary statistical testing to prevent variance inflation, but were retained for the overall geographical distribution analysis. To control for potential population stratification and background relatedness, Principal Component Analysis (PCA) was performed on genome-wide variants, and the top three principal components (PC1–PC3) were incorporated as covariates in the association regression model ( $y=\mu +\text{Haplotype}+\sum_{i=1}^{3}\beta iPCi+\epsilon$). In the model, *y* represents the observed phenotypic value (plant height or lignin content) of a given banana accession, μ is the model intercept (overall population mean), Haplotype is the fixed genetic effect of the candidate haplotype, PC_i_ denotes the i-th principal component (i=1,2,3) with its corresponding regression coefficient β_i_ to control for population stratification, and ϵ is the random residual error. To rigorously control for multiple testing across all 468 tested loci, both the conservative Bonferroni correction (*α* = 0.05/468, threshold: −log_10_ *P*>3.97) and the Benjamini–Hochberg False Discovery Rate (FDR *q* < 0.05) were applied to identify high-confidence candidate genes. Major haplotype differences were further evaluated using Tukey’s HSD test.

### 2.7 Linkage disequilibrium and geographical distribution analysis

Linkage disequilibrium (LD) blocks surrounding the lead candidates MaAP2ERF144 and MaAP2ERF407 were calculated using LDBlockShow (Dong et al., 2021), with the r^2^ threshold set at 0.8 to define stable inheritance blocks. Geographical coordinates for the 155 accessions were retrieved from the original collection records. The spatial distribution of major haplotypes was visualized on a global scale using the R-package ggplot2 and maps. Accessions were categorized by region of origin to evaluate the empirical geographical distribution of major haplotypes across regional germplasm collections.

### 2.8 Plant materials and height-gradient transcriptomic profiling

A hybrid population was established by crossing two wild banana accessions with polarized height traits to evaluate the genetic basis of plant height variation. The male parent was ‘Yunnan Xiaoguo wild banana’ (wild *Musa acuminata*, AA genome) which represents a dwarf resource with a plant height of 1.6 meters and the female parent was ‘South China wild banana’ (wild *Musa balbisiana*, BB genome) which exhibits a normal plant height of 3.1 meters. Six representative offspring from this population that exhibit a distinct height gradient ranging from 0.96 meters to 3.77 meters were selected for transcriptomic analysis.

Fresh pseudostem tissues were harvested from each of these individuals for total RNA extraction using the Plant RNA Kit from Tiangen. Library preparation and high throughput sequencing were performed on the Illumina platform. Gene expression levels were quantified as Transcripts Per Million (TPM). The raw RNA-sequencing datasets generated for the six height-gradient hybrid individuals have been deposited in the NCBI Sequence Read Archive (SRA) database under BioProject accession number PRJNA1529446 (SRA submission ID: SUB16485307, BioSample accession: SAMN63142814). In addition, the normalized transcript abundance (TPM matrix) for each sample is provided in **Supplementary Table S6** to support direct evaluation. Heatmaps were generated using Z score normalized TPM values via the Heatmap tool in TBtools to identify *MaAP2ERF* genes with expression patterns that positively or negatively correlate with the plant height.

## 3 Results

### 3.1 Pan-genomic identification and physicochemical characterization of the banana AP2/ERF family

To establish an exhaustive genomic framework, we performed a systematic pan-gene family analysis across eighteen diverse representatives of the Musaceae family. These genomes capture a broad taxonomic breadth, including the genera *Musa, Ensete*, and *Musella*. This diverse panel consists of wild species, landraces, and specialized cultivars, such as the fiber-producing *M. textilis*. By including multiple assemblies for specific species, we aimed to enhance the resolution of presence-absence variation (PAV) analysis. A total of 4,732 *MaAP2ERF* genes were identified across the eighteen genomes, from which 468 non-redundant representative members were characterized (**Supplementary Tables S1**, **S2**). These genes were sequentially designated as *MaAP2ERF1* through *MaAP2ERF468* based on their respective chromosomal positions. Then, the physicochemical properties of the family members were characterized to reveal the degree of structural conservation and evolutionary variation. The protein lengths ranged from 119 to 1015 amino acids with corresponding molecular weights spanning approximately 12.96 to 112.02 kDa (**Figures 1A, B**). Predicted subcellular localization analysis demonstrated that a vast majority of the members are localized to the nucleus, which is consistent with their typical roles as transcriptional regulators, while minor proportions were predicted in other compartments including chloroplasts, mitochondria, and cytoplasm (**Figure 1C**), suggesting specialized functional evolution.

**Figure 1:**
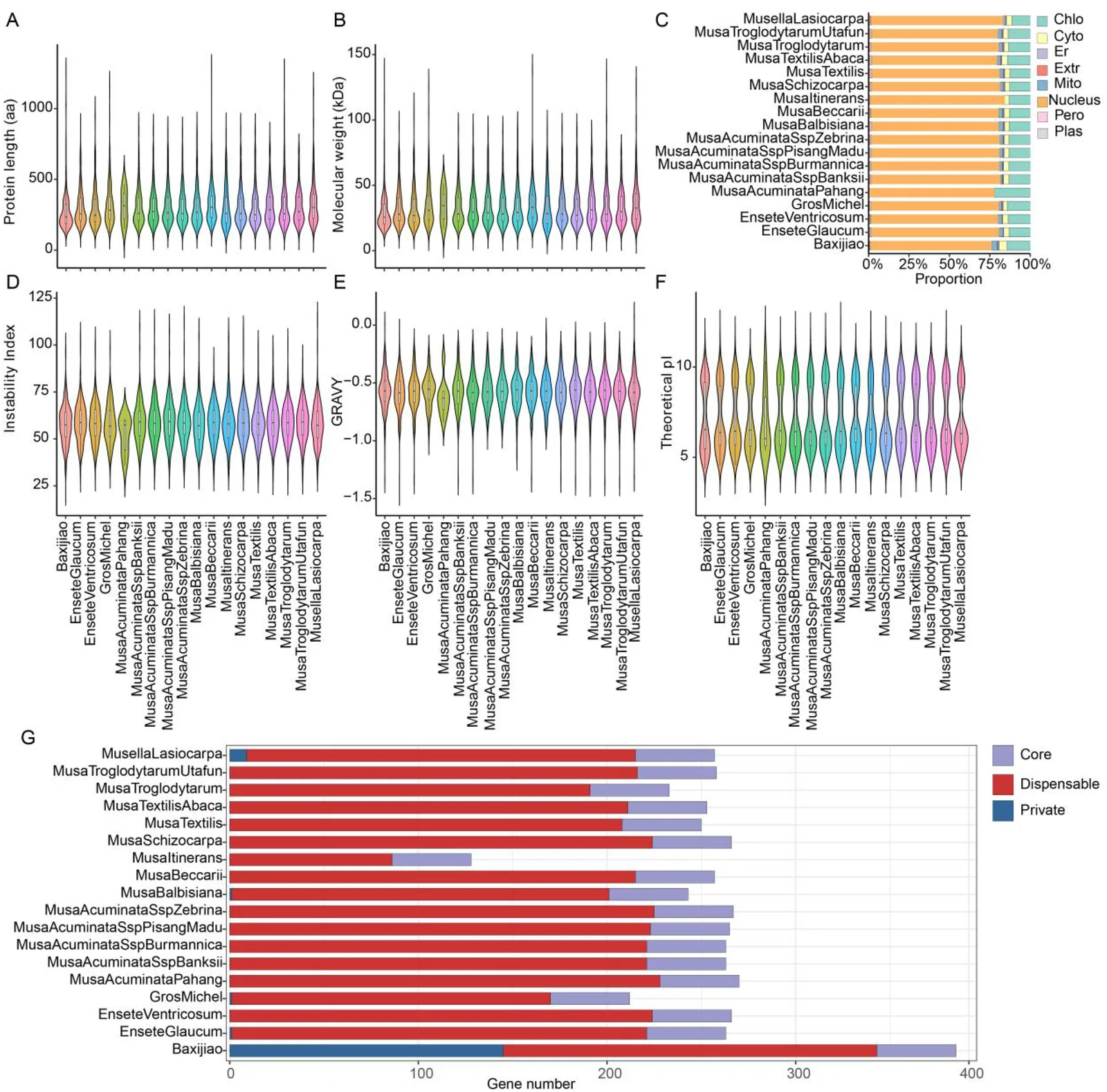
Pan-genomic identification and physicochemical properties of the *AP2/ERF* family in banana. **(A)** The distribution of protein lengths for the identified *MaAP2ERF* members across eighteen diverse banana genomes using violin plots. **(B)** The range of molecular weights for these proteins within each banana genome. **(C)** The proportion of predicted subcellular localizations for each genome. Chlo, Cyto, Er, Extr, Mito, Nucleus, Pero and Plas represent chloroplast, cytoplasm, endoplasmic reticulum, extracellular, mitochondria, cell nucleus, peroxisome and plasma membrane, respectively. **(D)** The instability index values which provide an assessment of protein stability across the eighteen banana genomes. **(E)** The distribution of GRAVY (Grand Average of Hydropathy) values to characterize the hydrophobicity and hydrophilicity of the family members. **(F)** The variation in theoretical isoelectric points within each specific genome. **(G)** The total gene number for each genome subdivided into core, dispensable, and private categories based on presence-absence variation analysis.

The stability and chemical properties were further evaluated using the instability index and GRAVY values. The instability index ranged from 22.75 to 98.24 (**Figure 1D**), indicating that the family comprises both structurally stable regulators (< 40) and dynamic proteins (> 40) susceptible to rapid cellular turnover. The GRAVY values varied from -1.342 to 0.007 (**Figure 1E**), with nearly all values being negative, confirming that the majority of MaAP2ERF members are hydrophilic, soluble transcription factors. Furthermore, the theoretical isoelectric points exhibited a broad distribution from 4.36 to 11.14 (**Figure 1F**). Finally, we resolved the genomic distribution of this family by categorizing the 468 non-redundant representative members based on their presence-absence variation across the eighteen genomes. Among these representative members, 42 genes were identified as core genes that are strictly conserved in all analyzed accessions. The remaining 426 members (91.03%) constituted the variable pan-gene pool, consisting of 269 dispensable genes present in multiple but not all accessions and 157 private genes unique to a single accession (**Figure 1G**). Collectively, these results provide a comprehensive genomic landscape of the *AP2/ERF* family and establish a powerful foundation for identifying the specific core and variable regulators that govern important agronomic traits such as plant height in banana.

### 3.2 Phylogenetic classification and the structural landscape of the banana *AP2/ERF* pan-gene family

To elucidate the evolutionary divergence and structure diversity of the *AP2/ERF* family at the pan-genomic level, we constructed a maximum likelihood phylogenetic tree using 468 representative members from banana and 121 known members from *Arabidopsis thaliana*. The *AP2/ERF* family was partitioned into four major subfamilies, which were *AP2, DREB, ERF*, and *Soloist*, based on the maximum likelihood tree (**Figure 2A**). Among the 468 representative members (**Supplementary Table S3**), the *ERF* group was identified as the most extensive subfamily with 268 genes, followed by the *DREB* group with 160 genes and the *AP2* group with 27 genes. The Soloist group contained the fewest representatives with only 13 members. These results indicate that the *ERF* subfamily serves as the primary driver of numerical expansion within the banana pan-gene family. Further investigation into the presence-absence variation (PAV) distribution within each subfamily (**Figure 2B**) revealed that the *ERF* group exhibits the highest degree of genomic variation through its majority of dispensable and private genes. Regarding the core genes, these were primarily concentrated in the *ERF* and *DREB* subfamilies, which indicated that members of these groups were subject to strong evolutionary constraints. The comparison of gene and protein structures revealed significant differences in complexity among the four subfamilies. Gene lengths showed a broad distribution particularly within the *ERF* group where some members reached up to 4,500 base pairs, whereas the *AP2*, *DREB* and *Soloist* subfamilies possessed significantly shorter gene lengths that were typically below 1,500 base pairs (**Figure 2C**). These trends were consistent with protein lengths where the *ERF* subfamily possessed the highest variation including large proteins such as *MaAP2ERF2* with 1,015 amino acids (**Figure 2D**). Analysis of intron structure further highlighted the unique complexity of the *ERF* subfamily. While the *AP2* and *DREB* members were relatively intron-poor, the *ERF* subfamily displayed the highest number of introns with some genes containing more than 15 introns (**Figure 2E**). Similarly, the total intron length was significantly higher in the *ERF* and *Soloist* groups than in the *AP2* and *DREB* subfamilies (**Figure 2F**). Collectively these findings indicate that the *ERF* subfamily has undergone the most extensive structural and genomic diversification during the evolution of the *Musa genus*.

**Figure 2:**
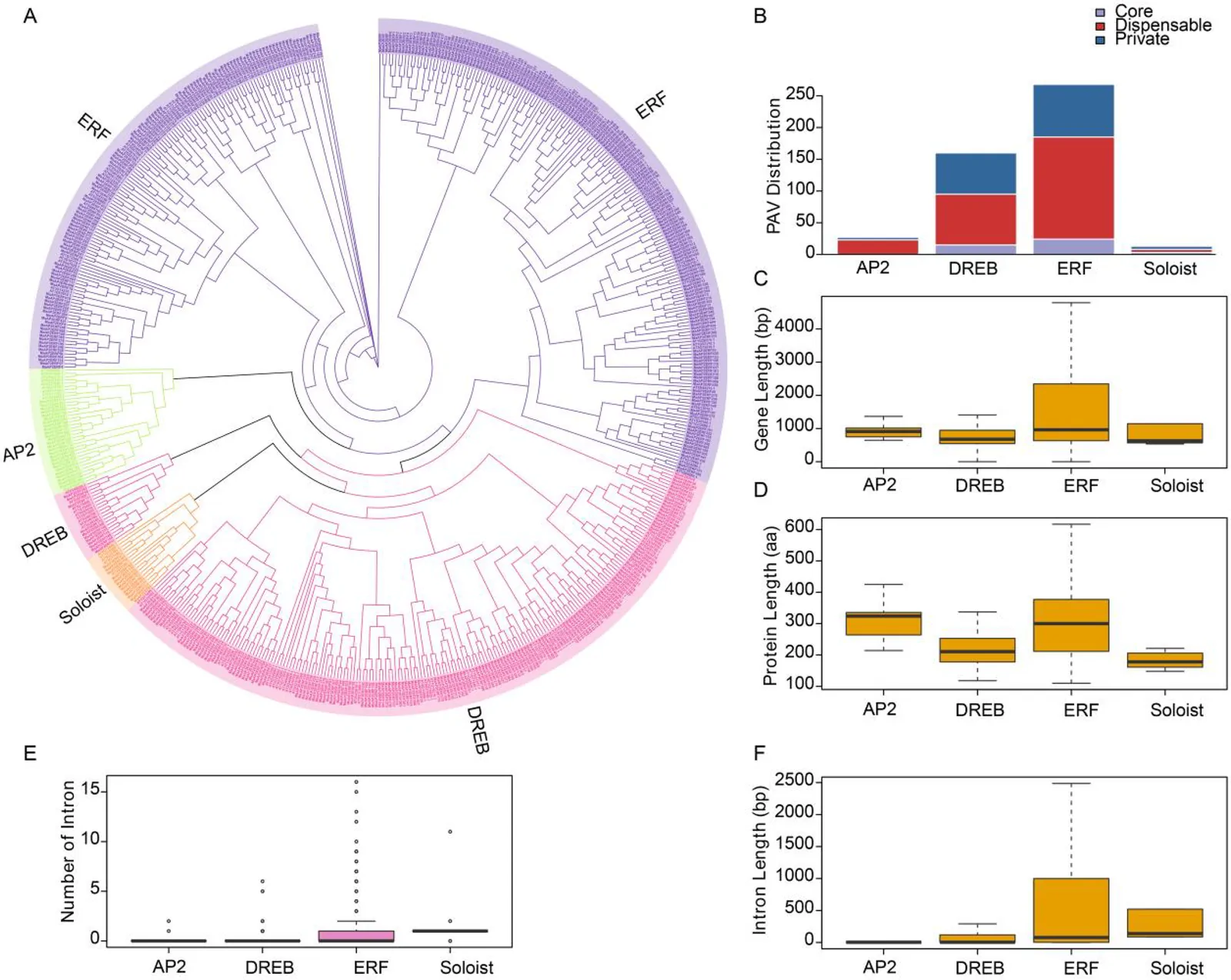
Phylogenetic relationships and structural characteristics of *AP2/ERF* subfamilies in the banana pan-genome. **(A)** Phylogenetic tree of 468 representative MaAP2ERF proteins and 121 *Arabidopsis thaliana AP2/ERF* members with method of maximum likelihood. Each clade distinguished by specific branch colors. **(B)** The distribution of core, dispensable, and private genes within each subfamily. **(C**, **D)** The variations in gene length **(C)** and protein length **(D)** among the four subfamilies using boxplots to highlight the structural range. **(E)** The distribution of intron numbers per gene across different subfamilies. **(F)** The variation in total intron length for each subfamily. The boxplots represent the median and interquartile ranges while the dots indicate individual outliers.

### 3.3 Evolutionary duplication modes and selection pressure of the *MaAP2ERF* pan-gene family

To elucidate the evolutionary mechanisms driving the diversification of the *AP2/ERF* pan-gene family and the selective pressures acting on its members, we analyzed gene duplication modes and the K_a_/K_s_ (Nonsynonymous to synonymous substitution) ratios which serve as a primary indicator of natural selection (**Supplementary Table S4**). Analysis of the duplication modes within the 468 non-redundant *MaAP2ERF* genes revealed that dispersed duplication contributed the largest numerical share to family expansion (43.38%, 203 genes), while WGD underpinned the conserved core repertoire (**Figure 3A**). Whole genome duplication or segmental events represented the second most frequent mode at 20.30 percent (97 genes), while singletons constituted 12.82 percent (60 genes). Proximal and tandem duplications each accounted for 11.54% of the representative members. Integration of duplication modes with PAV distribution revealed that all 157 private genes were generated through dispersed duplication (**Figure 3B**). The dispensable genes exhibited the most diverse evolutionary origins by utilizing all five duplication mechanisms in nearly equal proportions. Conversely, the 42 core genes were primarily derived from whole genome duplication or segmental events. This finding suggests that these specific genes were so essential that they have been preserved in the genome for millions of years without significant changes.

**Figure 3:**
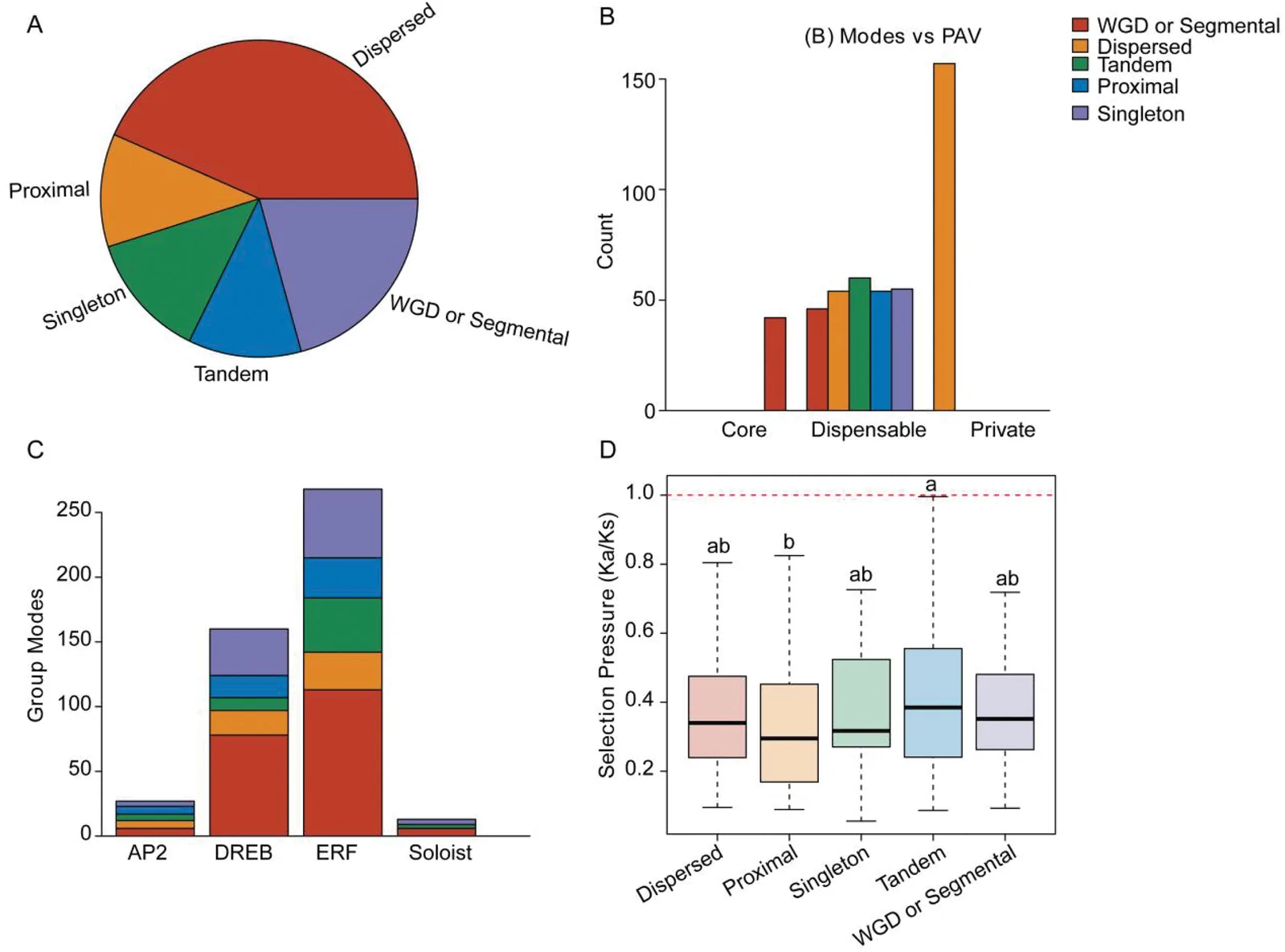
Gene duplication modes and selection pressure analysis of the banana *AP2/ERF* pan-gene family. **(A)** The proportion of five gene duplication types including dispersed, proximal, singleton, tandem, and whole genome duplication or segmental events. **(B)** The numerical distribution of these duplication modes across core, dispensable, and private gene categories. **(C)** The composition of duplication modes within the four subfamilies. **(D)** The boxplot of K_a_/K_s_ ratios for each duplication type where the horizontal line represents the median value. Different lowercase letters above the boxes denote statistically significant differences among duplication modes (*P* < 0.05, Kruskal-Wallis test followed by pairwise Wilcoxon rank-sum test with FDR adjustment).

Regarding the composition within each subfamily, the *AP2*, *DREB*, and *ERF* subfamilies all utilized the five duplication modes to reach their current sizes, while the *Soloist* subfamily was characterized by only three duplication types (**Figure 3C**). Selective pressure analysis demonstrated that the family is generally under strong purifying selection with the majority of K_a_/K_s_ ratios clustering between 0.2 and 0.5 (**Figure 3D**). Specifically, tandem duplications exhibited the highest median K_a_/K_s_ ratio of 0.39, while proximal duplications showed the lowest value of 0.29. The other three duplication modes, including dispersed, whole genome duplication, and singleton events, displayed intermediate median ratios of 0.34, 0.35, and 0.32 respectively. This comparison suggests that genes arising from tandem events may experience relatively relaxed selection pressure compared to other duplication types.

### 3.4 Distribution of selective pressures across the *MaAP2ERF* family

To evaluate how natural selection has influenced the 468 non-redundant representative members of the banana AP2/ERF family, we calculated the K_a_/K_s_ ratios for each gene across the eighteen genomes. The results demonstrated that the majority of family members are subject to strong purifying selection, with K_a_/K_s_ ratios clustering significantly below the neutral threshold of 1.0 and specifically within the 0.2 to 0.5 range (**Figure 4**; **Supplementary Table S4**). This peak distribution confirms that negative selection has been the primary evolutionary driver in maintaining the functional integrity of the *AP2/ERF* family across the *Musa* genus. Across the 353 calculable members, median Ka/Ks values remained tightly constrained across all subfamilies, ranging from 0.275 (AP2) and 0.336 (DREB) to 0.344 (ERF) and 0.350 (Soloist), with purifying selection dominating all lineages (>98% of valid genes). Similarly, core, dispensable, and private gene subsets displayed comparable evolutionary constraints, with median Ka/Ks ratios of 0.345, 0.338, and 0.337, respectively (**Supplementary Table S4**, Sheet ‘Summary Statistics’). While the family is mostly under negative selection, several genes with K_a_/K_s_ ratios over 1.0 were identified as candidates for positive selection and adaptive evolution. For example, MaAP2ERF141 (a WGD-derived member) exhibited the highest ratio of 1.199, followed by MaAP2ERF286 (1.192), MaAP2ERF168 (1.104), and MaAP2ERF248 (1.038). While these outliers demonstrate adaptive evolution, the overall family distribution (as reflected in **Figure 4**) confirms that the majority of members remain under strong purifying selection. Additionally, several members such as *MaAP2ERF114* and *MaAP2ERF254* displayed ratios of 0.957 and 0.944, suggesting a significant relaxation of selective constraints in these specific genes. These findings indicate that the banana *AP2/ERF* family is governed by a dual evolutionary strategy. While stringent purifying selection ensures the stability of core regulatory functions across the pangenome, the occurrence of gene-specific positive selection alongside reduced evolutionary constraints enables particular members to undergo adaptive innovation. This balance between conservation and change enables the family to maintain its essential biological roles, while simultaneously developing new functions in response to diverse environmental stimuli.

**Figure 4:**
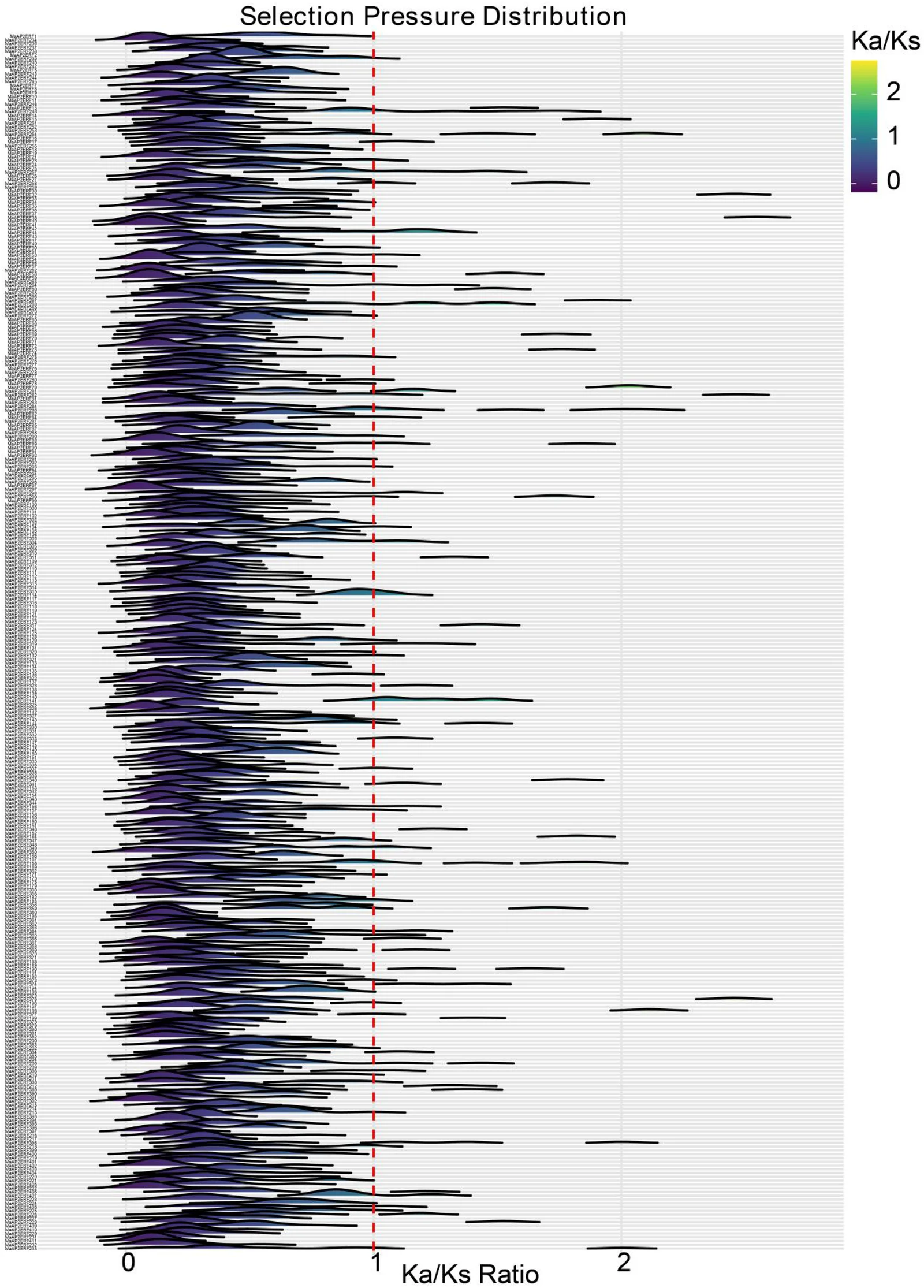
Distribution of selection pressure across 468 representative members of the banana *AP2/ERF* family. The ridgeline density plot illustrates the distribution of K_a_/K_s_ ratios for all non redundant members identified across the eighteen banana genomes. The color gradient from purple to yellow represents the magnitude of the K_a_/K_s_ values where purple signifies intense purifying selection. The red dashed line marks the 1.0 threshold for neutral selection. Data points below 1.0 indicate genes under negative selection, whereas values exceeding 1.0 identify candidates for positive selection and adaptive evolution. Detailed summary statistics (mean, median, IQR, and purifying/positive selection counts) partitioned by subfamily (ERF, DREB, AP2, Soloist) and PAV category (Core, Dispensable, Private) are provided in **Supplementary Table S4** (Sheet: Summary Statistics). Outliers exhibiting signatures of positive selection (Ka/Ks>1.0) include *MaAP2ERF141* (1.199), *MaAP2ERF286* (1.192), *MaAP2ERF168* (1.104), and *MaAP2ERF248* (1.038).

### 3.5 Integration of pan-family *MaAP2ERF* analysis with published phenotypic data identifies candidate genes associated with plant height variation

To systematically identify the potential determinants of plant architecture within the 468-member *MaAP2ERF* transcription factor family, we integrated the pan-family genomic data with previously published plant height phenotypic datasets through a haplotype-based association study. To ensure statistical robustness and capture rare but significant phenotypic variations, only haplotypes represented by at least three accessions (N≥3) were included in the ANOVA and phenotypic comparisons (marked as In_Statistic = True in **Supplementary Table S5**). This threshold was specifically selected because, in clonal crops like banana and diverse wild germplasm, elite alleles associated with extreme phenotypes are often concentrated in a few rare haplotypes. Setting the threshold at N≥3 allows for the identification of these high-value genetic determinants without losing important variation due to overly stringent filtering. The pangenome-wide association mapping across 468 members identified several significant loci. To minimize false positives, a stringent Bonferroni-corrected threshold (−log_10_ *P*>3.97; *α* = 0.05/468) was applied (**Figure 5A**). Under this criterion, four high-confidence candidates were identified: *MaAP2ERF407*, *MaAP2ERF144*, *MaAP2ERF84*, *and MaAP2ERF258* (**Supplementary Table S5**). Among these, *MaAP2ERF407* and *MaAP2ERF144* were prioritized as the lead regulators for further investigation due to their superior statistical strength. *MaAP2ERF407* (located on Chromosome 11) emerged as the most significant candidate locus, exhibiting a *P*-value of 3.96×10^−6^ (−log_10_ *P* = 5.402) and the largest effect size of 285.2. *MaAP2ERF144* (Chromosome 06) was identified as another primary locus, with a *P*-value of 7.57×10^−6^ (−log_10_ *P* = 5.121) and an effect size of 268.8. Linkage disequilibrium (LD) analysis confirmed stable inheritance blocks surrounding these two primary loci (**Figure 5B**).

**Figure 5:**
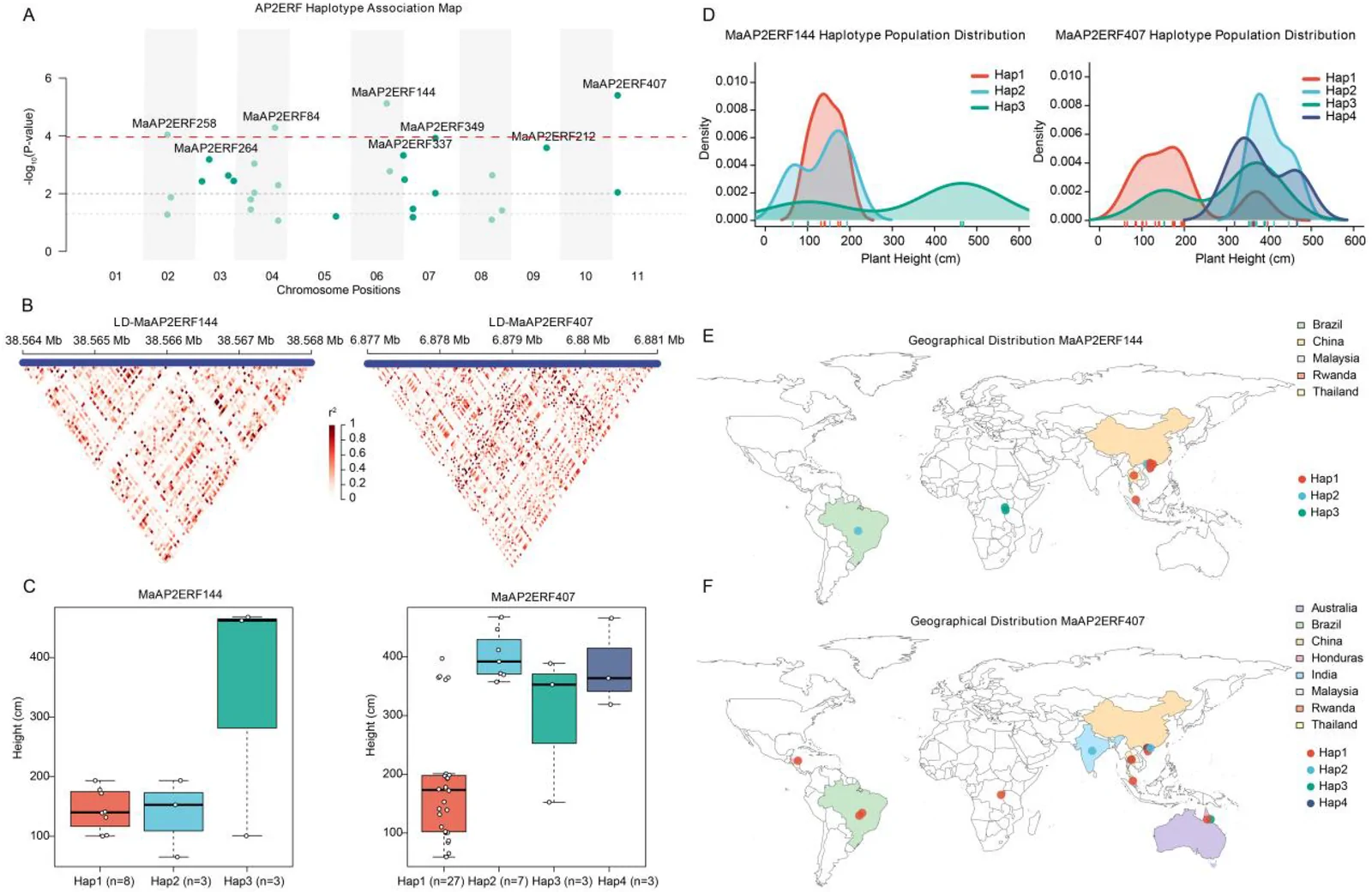
Haplotype-based association mapping and geographical characterization of *MaAP2ERF* family members based on published plant height data. **(A)** Manhattan plot of plant height associations focusing on *MaAP2ERF* members identified from the banana pan-gene family. **(B)** LD heatmaps for lead candidates *MaAP2ERF144* and *MaAP2ERF407* showing significant haplotype blocks (r^2^>0.8). **(C)** Haplotype-phenotype boxplots illustrating significant height stratification. Each point represents a banana accession. Horizontal black lines and white circles within the boxes indicate the median and mean values, respectively. **(D)** Population distribution profiles showing bimodal density shifts driven by elite haplotypes. **(E, F)** Global geographical distribution of major haplotypes across 103 accessions. Background colors represent source countries. Point colors correspond to haplotypes in panel **(C)**.

Detailed analysis of the published population data revealed that allelic variations in these two genes significantly contribute to plant height diversity (**Figures 5C, D**). For *MaAP2ERF144*, high genetic diversity resulted in 37 different haplotypes across the population. Our statistical analysis focused on the three largest groups which are Hap1 with 8 accessions, Hap2 with 3 accessions, and Hap3 with 3 accessions. This selection followed our rule of including only haplotypes with at least 3 samples for reliable testing. Although some tall varieties were in rare groups and were excluded from the ANOVA, the clear height differences among these main haplotypes prove that *MaAP2ERF144* is a key candidate gene associated with plant height variation. Accessions harboring Hap3 exhibited a stark height advantage, with representative samples such as 01R0104 (467.81 cm) and 01R0227 (462.11 cm) demonstrating superior growth compared to the population average. In contrast, Hap1 (such as 01R0011, 139.14 cm) and Hap2 (such as 01R0153, 65.27 cm) were predominantly associated with dwarf or semi-dwarf phenotypes. For *MaAP2ERF407*, the population was divided into four haplotypes. Hap2 and Hap4 were identified as height-increasing alleles. For example, sample 01R0231 (467.66 cm) carries Hap2, while 01R0233 (465.89 cm) carries Hap4. Conversely, Hap1 represents a widespread short-height allele, exemplified by samples 01R0050 (100.32 cm) and 01R0026 (59.07 cm).

The spatial distribution of these haplotypes across global accessions revealed distinct geographical partitioning (**Figures 5E, F**). For *MaAP2ERF144*, Hap1 is overwhelmingly dominant in East Asia (China), characterizing the typical architectural profile of regional cultivars. In contrast, the superior Hap3 is primarily concentrated in Southeast Asian regions (Malaysia, Thailand) and Oceania (Australia), and is notably also carried by accessions from East Africa (such as 01R0082 and 01R0227 from Rwanda). Similarly, height-increasing variations for *MaAP2ERF407* were identified in diverse origins, including South Asia (Hap2 in India) and Southeast Asia (Hap4 in Thailand). This geographical partitioning suggests that these specific *MaAP2ERF* haplotypes are differentially distributed across regional banana germplasms.

To provide direct biological evidence for these genetic associations, we further validated the lead candidates using transcriptomic profiling (RNA-seq) across a hybrid population (‘Yunnan Xiaoguo’ × ‘South China wild banana’) exhibiting a sharp height gradient from 0.96 m (Shortest, Height_1) to 3.77 m (Tallest, Height_6) (**Supplementary Figure S1**; **Supplementary Table S6**).

Notably, *MaAP2ERF144* exhibited peak expression (Z-score > 2.0) in the shortest individual (Height_1), with expression levels decreasing progressively as plant height increased across the offspring (**Supplementary Figure S1**). This strong negative correlation between transcript abundance and vertical growth suggests that MaAP2ERF144 may function as a negative regulator or growth repressor of pseudostem elongation. While *MaAP2ERF407* showed relatively stable basal expression in this specific hybrid background, its robust genetic association with height in the broader population highlights its significance as an important candidate factor in plant height variation.

In summary, while pangenome-wide analysis identifies MaAP2ERF144 and MaAP2ERF407 as lead candidate genes associated with plant height, our transcriptomic validation further pinpoints *MaAP2ERF144* as the most critical candidate due to its strong expression-height correlation. These findings reveal the geographical distribution of height-related alleles and underscore MaAP2ERF144 as a primary functional candidate for molecular-guided crop ideotype improvement.

## 4 Discussion

### 4.1 Pan-genome analysis uncovers massive hidden variation and high plasticity within the banana *AP2/ERF* transcription factor family

The comprehensive identification of 468 non-redundant *MaAP2ERF* representative members across eighteen genomes provides a significant update to the current understanding of this family in banana. Previous investigations based on single reference assemblies identified 265 and 318 *AP2/ERF*-encoding genes in *M. acuminata* and *M. balbisiana*, respectively (Lakhwani et al., 2016). Across the eighteen genomes analyzed in this study, individual accessions harbored an average of approximately 263 members (4,732 total genes across 18 genomes), closely mirroring these earlier single-genome estimates. The substantially larger pan-family catalog of 468 non-redundant representative members thus underscores the cumulative gene diversity captured by multi-accession sampling across diverse Musaceae lineages, rather than an elevated gene complement within any individual genome. This pattern is directly substantiated by our presence-absence variation (PAV) analysis, which revealed that the banana *AP2/ERF* family consists of a relatively small conserved backbone of 42 core genes (8.97%), while the vast majority (426 genes, 91.03%) are classified as dispensable (269 genes) or private (157 genes). This high proportion of variable genes is consistent with previous pan genomic findings in soybean (Gao and Zhao, 2025) and *Brassica napus* (Xue et al., 2026). These results illustrate that while the basal per-genome repertoire of the *AP2/ERF* family remains relatively stable, a single reference genome inevitably fails to capture the extensive lineage-specific variation across germplasms. Establishing a multi-genome framework is therefore essential to uncover the hidden genomic plasticity that has arisen during Musa diversification.

When evaluated in a broader monocot evolutionary context, the banana *AP2/ERF* gene family displays a prominent lineage-specific expansion. In single reference genomes of representative monocots, *AP2/ERF* family sizes typically range from approximately 100 to 200 members, as exemplified by pineapple (97 genes; Zhang et al., 2021), sorghum (158 genes; Mathur et al., 2020), rice (163 genes; Sharoni et al., 2011), and maize (214 genes; Zhang et al., 2022). At the pan-genomic scale, a panel of 76 accessions in barley resolved into 383 orthologous gene groups (Bai et al., 2025). In comparison, the Musaceae genomes analyzed here show a consistently larger family size: individual assemblies harbor an average of 263 members, and the combined pan-genome captures 468 non-redundant representative members.

This evolutionary expansion can be understood through the distinct genomic history and specialized growth habit of the genus *Musa*. First, the *Musa* lineage experienced three ancient rounds of whole-genome duplication (D'Hont et al., 2012), which provided an extensive duplicated genomic backbone that retained more transcription factor copies than many other monocot clades. Second, bananas are giant perennial monocotyledonous herbs with massive pseudostems, distinct from the growth habits of typical annual grasses or rosette monocots. Rapid vertical extension and the need for mechanical stability to resist tropical storms require coordinated transcriptional control over both cell elongation and secondary cell wall lignification. The retention and functional divergence of duplicated AP2/ERF regulators likely supported these demanding architectural adaptations. Thus, ancient WGD anchored the conserved regulatory foundation of the family, while ongoing dispersed duplications continually expanded the *AP2/ERF* repertoire across wild relatives and cultivars. Ultimately, this comprehensive genomic landscape provides a vital resource for molecular breeding by offering the precise information needed to promote plant growth and environmental resilience across the entire species.

### 4.2 WGD and dispersed duplications drive the evolutionary stability and functional diversification of banana *MaAP2ERF* genes

The structural diversity and lineage-specific expansion of gene families are shaped by distinct duplication mechanisms with divergent evolutionary trajectories. In this study, our comparative duplication analysis revealed that dispersed duplication contributed the largest numerical share to family expansion, accounting for 43.38% (203 genes) of the representative members and generating all 157 private genes (**Figure 3**). Dispersed duplication is often associated with transposable element activity and illegitimate recombination, which physically translocate gene copies away from their ancestral genomic locations (Song and Cao, 2017). By moving paralogs into novel genomic environments, dispersed duplication enables these duplicated copies to escape ancestral cis-regulatory constraints and acquire distinct expression profiles or neofunctionalized roles (Chen et al., 2023). This pattern aligns with findings in the Cucurbitaceae *bZIP* pan-gene family (Sun et al., 2025), explaining how peripheral gene copies provide mutational flexibility to facilitate environmental adaptation.

However, this numerical expansion must be interpreted alongside the foundational role of whole-genome duplication (WGD). Crucially, WGD and segmental duplication events accounted for all 42 core genes (100%) strictly conserved across all 18 analyzed Musaceae genomes. This observation is consistent with the established evolutionary history of the *Musa* lineage, which underwent three ancient rounds of whole-genome duplication that provided the primary genetic templates for fundamental developmental regulators (D'Hont et al., 2012). In plant transcriptional networks, WGD typically duplicates entire regulatory pathways simultaneously, thereby maintaining the dosage balance of critical macromolecular complexes. The strict preservation of these WGD-derived core members under intense purifying selection (Ka/Ks clustering between 0.2 and 0.5) underscores their essential, non-redundant functions in directing core developmental programs and basic plant architecture.

Therefore, rather than attributing family evolution to a single duplication mode, the banana AP2/ERF family is characterized by a cooperative, two-tiered evolutionary mechanism: ancient WGD in the *Musa* lineage established the conserved and indispensable core functional foundation, while subsequent dispersed duplications acted as the primary engine driving numerical expansion and lineage-specific diversification.

### 4.3 Divergent selection patterns across the *MaAP2ERF* family provide a genomic perspective for the targeted improvement of banana architecture

The distribution of selective pressures within the *MaAP2ERF* family reflects a sophisticated balance between functional preservation and adaptive innovation. Our calculation of K_a_/K_s_ ratios revealed that while the majority of members are under rigorous purifying selection to maintain core developmental programs, specific genes exhibit signatures of positive selection or relaxed constraints (**Figure 4**). The predominance of K_a_/K_s_ values below 0.5 for core genes emphasizes their essential roles in banana survival (Hurst, 2002). However, the identification of members like *MaAP2ERF141* with ratios exceeding 1.0 (specifically 1.199) indicates that these specific members have evolved specialized capabilities through adaptive diversification. The identification of *MaAP2ERF141*, a dispensable gene arising from WGD, as the member with the highest Ka/Ks ratio provides a nuanced view of the evolutionary stability of the banana pan-genome. While the collective data (**Figure 3D**) confirms that WGD genes are typically subject to the most intense purifying selection to maintain structural integrity, the positive selection observed in *MaAP2ERF141* suggests that specific duplicated copies can escape these constraints. This highlights a dual evolutionary mechanism where WGD provides both a stable framework and the raw material for rapid adaptive innovation through potential neofunctionalization. This relaxation of selective pressure is particularly evident in the dispensable and private gene sets which often show higher K_a_/K_s_ values than core genes. Such a pattern has been observed in the pan-genomes of *Brassica napus* L and barley, where variable genes are allowed more mutational flexibility to facilitate adaptation to diverse environmental contexts or specific selection targets (Wang et al., 2025; Bai et al., 2025). The discovery of distinct selection patterns across different gene sets helps explain the genetic diversity found in banana. These insights allow for more precise genetic modification by identifying which family members are most open to functional changes without disrupting important biological processes. This strategy helps breeders target specific traits more safely and reduces the risk of causing unwanted growth problems. Consequently, this work provides a reliable foundation for advancing banana production and serves as a guide for studying other important horticultural species.

### 4.4 Haplotype divergence of *MaAP2ERF* members governs architectural plasticity and geographical diversity in banana

Plant height is a complex agronomic trait primarily governed by cell elongation and mechanical support. In this study, by integrating a pan-family analysis of 468 MaAP2ERF members with population-scale phenotypic data, we identified *MaAP2ERF407* and *MaAP2ERF144* as the primary candidate genes associated with banana height variation (**Figure 5**). Previous investigations in model species such as rice and *Arabidopsis* have firmly established the *AP2/ERF* family as a critical determinant of plant height and architectural development by modulating key growth-related hormone pathways (Licausi et al., 2013). In banana, while prior research has focused on *MabHLH30* as primary height regulators (Li et al., 2025b), our findings suggest that specific MaAP2ERF transcription factors represent promising candidate regulators of banana architecture. The significant effect sizes of 285.2 for *MaAP2ERF407* and 268.8 for *MaAP2ERF144* indicate that these members explain a substantial portion of the phenotypic variance in height across the *Musa* pan-genome, offering more potent targets for architectural modification than downstream metabolic genes.

A distinctive feature of our results is the dual association of *MaAP2ERF407* with both plant height (-log_10_ *P* = 5.402) and lignin content (-log_10_ *P* = 2.044). This pleiotropic effect suggests a coordinated regulatory mechanism between vertical growth and the biosynthesis of the secondary cell wall. In giant herbaceous plants like banana, rapid elongation must be synchronized with increased mechanical support to prevent lodging. Previous literature has documented the role of *ERF* transcription factors in regulating the lignin biosynthetic pathway in Populus (Wessels et al., 2019). However, the identification of a single *MaAP2ERF* member (*MaAP2ERF407*) displaying dual associations with both height and lignification provides a novel genetic basis for the ‘mechanical-height’ trade-off in banana.

The geographical distribution of *MaAP2ERF* haplotypes (**Figures 5E, F**) reveals distinct allelic patterns across the 103 georeferenced accessions in our collection. For instance, short-height alleles (*MaAP2ERF144*-Hap1 and *MaAP2ERF407*-Hap1) predominate among East Asian accessions, whereas height-increasing alleles (*MaAP2ERF144*-Hap3 and *MaAP2ERF407*-Hap2/4) are predominantly found in accessions originating from Southeast Asia, Oceania, South Asia, and East Africa.

However, we emphasize that this observed geographical partitioning must be interpreted with caution. A substantial limitation of the current population panel is ascertainment and sampling bias: accessions from East Asia (primarily southern China) constitute a major proportion of the collection, reflecting regional repository availability rather than a balanced global survey of *Musa* biodiversity. Furthermore, commercial bananas are predominantly sterile, clonally propagated cultivars. In such clonal systems, widespread vegetative dissemination of specific elite cultivars (such as dwarf Cavendish derivatives in East Asia) can generate strong founder effects that mimic the signatures of regional adaptive selection. Without comprehensive demographic modeling and balanced sampling of indigenous wild populations across the native ranges of *Musa*, we cannot eliminate the possibility that this regional distribution primarily reflects historical germplasm movement, agricultural preference, and sampling availability rather than ancient evolutionary selection.

Nevertheless, the identification of identical height-associated alleles across trans-continental lineages demonstrates that these allelic variants are phylogenetically widespread and not localized artifacts of Chinese germplasm. For example *MaAP2ERF144*-Hap3, it was consistently detected in wild and cultivated accessions from Thailand, Australia, and Rwanda. Combined with independent validation in our biparental hybrid population (**Supplementary Figure S1**), these findings confirm the functional potential of *MaAP2ERF144* in governing banana pseudostem elongation, while providing an empirical map of allele availability for future regional breeding programs. We note that the evidence in this study is primarily associational and correlative. Transgenic validation was not performed due to the long regeneration cycle of banana. In the future, reverse-genetics studies such as CRISPR/Cas9 knockout and transgenic complementation will be needed to confirm the direct biological functions of *MaAP2ERF144* and *MaAP2ERF407.* Furthermore, future studies integrating cutting-edge structural biology tools such as AlphaFold3 to model the 3D conformations and protein-DNA binding interfaces of these core candidates and elite allelic variants will provide deeper structural insights into their transcriptional regulatory mechanisms.

## Data Availability

The original contributions presented in the study are included in the article/**Supplementary Material**. Further inquiries can be directed to the corresponding authors.
