# Supplementary material for: Pan-genome analysis of the AP2/ERF gene family and its functional characterization in banana plant height
Source: Front Plant Sci. 2026 Oct 19;17:1944831. doi: 10.3389/fpls.2026.1944831 (PMC13724342; doi:10.3389/fpls.2026.1944831)
Supplement: Supplementary file 1 [file Table7.docx]

**Figure S1. Expression heatmap of lead *MaAP2ERF* candidates across a hybrid population with a plant height gradient.**

The heatmap displays Z-score normalized TPM (Transcripts Per Million) values for *MaAP2ERF144* and *MaAP2ERF407* in six hybrid offspring. Samples are arranged phenotypic order from the shortest (Height_1, 0.96 m) to the tallest (Height_6, 3.77 m). Red color indicates high expression, and blue color signifies low expression.

The heatmap illustrates the relative transcript abundance of *MaAP2ERF144* and *MaAP2ERF407* in pseudostem tissues of six hybrid offspring. The color scale represents Z-score normalized TPM (Transcripts Per Million) values, calculated row-wise for each gene to highlight relative expression changes across the phenotypic gradient. Samples are arranged in phenotypic order from the shortest (Height_1, 0.96 m) to the tallest (Height_6, 3.77 m). In the color bar, positive values (red) indicate expression levels higher than the mean across all samples, while negative values (blue) indicate expression levels lower than the mean. A Z-score of 0 (gray/tan) represents the average expression level for that specific gene.
