# Supplementary material for: Pan-genome analysis of the AP2/ERF gene family and its functional characterization in banana plant height
Source: Front Plant Sci. 2026 Oct 19;17:1944831. doi: 10.3389/fpls.2026.1944831 (PMC13724342; doi:10.3389/fpls.2026.1944831)
Supplement: Supplementary file 2 [file Table8.docx]

import pandas as pd

import gzip

import os

import sys

\# --------------------------------

\# Project: Banana AP2/ERF Pan-geno

\# Script: Functional Haplotype Ass

\# Description: This script process

\#	define haplotypes b

\#	and promoter region

\# --------------------------------

\# Define input/output directories

INPUT_VCF_DIR = "Priority_Genes_VC

NR_GENE_LIST = "Final_Priority_Can

OUTPUT_DIR = "Hap_Results_Final_定

\# Create output directory if not e

if not os.path.exists(OUTPUT_DIR):

os.makedirs(OUTPUT_DIR)

def define_functional_haplotypes()

"""

Identifies major haplotypes fo

filtering for functional varia

"""

\# Load the prioritized non-red

if not os.path.exists(NR_GENE_

print(f"Error: Required fi

return

gene_targets = pd.read_csv(NR_

gene_ids = gene_targets['MaID'

print(f"Starting haplotype ana

for gene_id in gene_ids:

vcf_path = os.path.join(IN

if not os.path.exists(vcf_

continue

samples = []

genotype_matrix = []

\# 1. Parse VCF and extract

\# Regions include coding s

with gzip.open(vcf_path, '

for line in vcf:

if line.startswith

if line.startswith

samples = line

continue

cols = line.strip(

info_field = cols[

\# Filter for varia

\# Criteria: Missen

is_functional = an

if is_functional:

\# Extract the

\# Handles both

gt_row = [s.sp

\# Quality Cont

if gt_row.coun

genotype_m

if not genotype_matrix:

continue

\# 2. Convert to Dataframe

df_hap = pd.DataFrame(geno

\# 3. Concatenate individua

df_hap['HapString'] = df_h

df_hap = df_hap.reset_inde

\# 4. Filter haplotypes by

\# Ensures statistical robu

hap_counts = df_hap['HapSt

major_hap_strings = hap_co

\# Mapping haplotype string

hap_name_map = {string: f"

df_hap['HapName'] = df_hap

df_hap['In_Statistic'] = d

\# 5. Export processed data

output_path = os.path.join

df_hap[['SampleID', 'HapNa

print(f"Processed {gene_id

if __name__ == "__main__":

define_functional_haplotypes()
